# A gestational ketogenic diet alters maternal metabolic status as well as offspring physiological growth and brain structure in the neonatal mouse

**DOI:** 10.1186/1471-2393-13-198

**Published:** 2013-10-29

**Authors:** Dafna Sussman, Jacob Ellegood, Mark Henkelman

**Affiliations:** 1 Department of Medical Biophysics, Faculty of Medicine, University of Toronto, Toronto, Canada; 2 Mouse Imaging Centre (MICe), The Hospital for Sick Children, Toronto, Canada

**Keywords:** Neonatal brain development, Ketogenic diet, Low-carbohydrate diet, Mouse imaging, Magnetic resonance imaging (MRI), CD-1 mouse

## Abstract

**Background:**

The use of the ketogenic diet (KD) among women of child-bearing age has been increasing, leading to increased interest in identifying the diet’s suitability during gestation. To date, no studies have thoroughly investigated the effect of a gestational KD on offspring growth. Since ketones have been reported to play a role in cerebral lipid and myelin synthesis, it is particularly important to investigate the diet’s impact on brain anatomy of the offspring.

**Methods:**

To fill this knowledge gap we imaged CD-1 mouse neonates whose mothers were fed either a standard diet (SD) or a KD prior to and during gestation. Images were collected at postnatal (P) 11.5 and 21.5 using Magnetic Resonance Imaging (MRI). Maternal metabolic status was also tracked during lactation, by following their body weight, blood glucose, ketone, cholesterol, and triglyceride concentrations.

**Results:**

The KD dams exhibit a significant reduction in maternal fertility and litter size, as well as a high risk of developing fatal ketoacidosis by mid-lactation. To increase survival of the KD dams and offspring, fostering of P2.5 pups (from both KD and SD litters) by SD-foster dams was carried out. This resulted in stabilization of blood ketones of the KD dams, and aversion of the fatal ketoacidosis. We also note a slower and smaller weight loss for the KD compared with the SD dams. The average fostered KD pup exhibits retarded growth by P21.5 compared with the average fostered SD pup. An anatomical comparison of their brains further revealed significant structural differences at P11.5, and particularly at P21.5. The KD brain shows a relative bilateral decrease in the cortex, fimbria, hippocampus, corpus callosum and lateral ventricle, but a relative volumetric enlargement of the hypothalamus and medulla.

**Conclusion:**

A gestational ketogenic diet deleteriously affects maternal fertility and increases susceptibility to fatal ketoacidosis during lactation. Prenatal and early postnatal exposure to a ketogenic diet also results in significant alterations to neonatal brain structure, and results in retarded physiological growth. These alterations could be accompanied by functional and behavioural changes in later postnatal life.

## Background

The low-carbohydrate, high-fat ketogenic diet (KD) has been shown to be effective in managing a variety of diseases, including epilepsy, obesity, and disorders of the central nervous system [1-5]. It is also used for body-building purposes, and as a general lifestyle diet among healthy adults. This broad and growing use by adults, and particularly by women of child-bearing age, raises a concern about its use during gestation and its potential effects on offspring growth and brain development.

Studies on adult humans and rodents have shown that sparse carbohydrate and glucose availability initiates a process of ketogenesis that produces ketones, which all organs – including the brain – can use for energy [6,7]. During gestation, since the embryo’s capability to carry out ketogenesis is very limited, it relies on ketone supply from maternal circulation. Ketone supply is modulated by the carriers Monocarboxylate Transporters (MCTs) [8,9]. Studies using non-ketogenic gestational diets report that MCT expression in the placenta decreases at the end of gestation [10], implying restricted ketone availability to the fetus. This observation seems contrary to other studies that reported an increase in ketone utilization enzymes within the brain during the second half of gestation [6]. This increase in ketone utilization enzymes was speculated to facilitate lipid and myelin (white-matter) synthesis [11], implying greater availability would be favourable for the developing brain. A similar conclusion could be drawn from studies linking ketones and cerebral protein synthesis [11,12]. Yet, while ketones may be important metabolic substrates, an excessive supply to fetal brain can also damage nucleotide bio-synthesis, impairing nucleic acid production and brain growth [13]. Hence, it is important to determine whether a gestational KD indeed alters early brain development.

Investigations of nutritional effects on growth and brain development are often conducted on the mouse due to the high genetic homology it shares with humans. However, unlike the human brain, the mouse brain continues in its rapid growth during the first few weeks post-natally, until weaning, at Post-natal day (P) 21.5 [14,15]. Therefore, investigation of nutritional effects on early anatomical brain development using the mouse model must consider the first three weeks postnatally.

In our previous study we started investigating the physiological consequences of a gestational KD on the offspring [16]. That study focused on the embryonic period and revealed embryonic growth retardation as well as alterations in brain structure just prior to parturition. The current study focuses on the next developmental period - the neonatal period - and investigates the implication of a gestational KD for the neonatal mouse. The neonatal mouse brain previously exposed to a maternal gestational Standard Diet (SD) is compared to that exposed to a maternal gestational KD. This investigation is carried out using 3D magnetic resonance imaging (MRI). Conventionally, T1 and T2 weighted sequences are employed for such investigation. However, these sequences provide limited grey to white matter contrast in the neonatal brain, due to the sparse myelin at that period. As a result, image alignment and registration accuracy is also compromised, leading to poor structural assessment. Instead, and to improve the amount of anatomical information gathered from neonatal brain imaging, we utilized a Diffusion Tensor Imaging (DTI) sequence, which provides the regional grey/white matter contrast, as previously employed by Zhang et al. (2006) [17].

## Methods

Six-week-old CD-1 male and female mice were weight-matched and randomly assigned to either a control group on a SD, or a study group on a KD. Mice were fed their respective diets for a period of 30 days, and then naturally mated by setting up a single male with a single female from their respective group. The morning newly-born pups were observed was considered day 0.5 of post-natal life (P0.5). Post parturition, pups were perfused and their skull and brain were extracted at one of two time-points: P11.5 and P21.5, corresponding to the end of the rapid brain growth and soon after the onset of the period of rapid myelination, respectively [14,18].

At the beginning of the study we discovered that the KD dams were unable to lactate properly, as described in the results. The protocol was altered to ensure the viability of the KD dams and pups until weaning. Instead of remaining with their mothers, the KD pups were separated from their biological mothers at P2.5. Arbitrarily selected ones were then fostered by lactating SD foster dams whose pups were at the same age as the KDs. The control group was also fostered in a similar manner. The SD and KD pups who were not fostered, were euthanized.

### Diet

The SD and KD were both manufactured by Harlan (Madison, WI). The SD consisted of 5% fat, 76.1% carbohydrate, and 18.9% protein, and wt/wt (Teklad diet no. TD.2918), providing 3.1 Kcal/gr. The KD consisted of 67.4% fat, 0.6% carbohydrate, and 15.3% protein wt/wt, which is equivalent to a 4:1 ratio by weight of fat to combined protein and carbohydrate (Teklad diet no. TD.96355) [19]. It provided 6.7 Kcal/gr, which is more than double the energy density of the SD. All mice were allowed unlimited access to their food and water prior to mating, during gestation, as well as post parturition.

### Animals

All animal procedures in this study were approved by the Animal Care Committee of the Toronto Centre for Phenogenomics (TCP), and were carried out in accordance with the standards of the Canadian Council on Animal Care. During lactation, and between P2.5 and P20.5, maternal body weight, blood glucose and *β*−ketones (*β*−*hydroxybutyrate*) were measured every other day. Glucose and ketone concentrations were measured with an Abbott “Precision Xtra” glucometer which required 0.6-1.5 *μ*L of whole-blood per test [20], drawn from the tail vein. Body weight of the pups was also measured at the same frequency and time period, and served for assessing overall growth between P2.5 and P20.5, which corresponded to weaning.

Cholesterol and triglycerides were tested at P2.5 and P21.5 in the SD and KD dams who were separated from their litters at P2.5. This test required a combined whole-blood volume of 190 *μ*L, collected from the saphenous vein, and was used for assessing the impact of the ketogenic diet on serum fat and body energy storage.

Adoption of KD and SD pups by SD foster dams was carried out by mixing the adopted pups in wet and soiled bedding from the foster dam’s cage. This ensured that they were similar in scent to the biological pups. As well, approximately half of the biological pups were kept, and their tails were clipped, for easier distinction between them and the adopted pups. The number of adopted pups was such that the total new litter size did not exceed a 30% increase compared to the biological litters size. Following adoption, cages were not disturbed for 48 hours. Any biological or adopted pups that did not undergo fostering by an SD foster dam were euthanized.

The statistical analysis utilized Analysis of Variance (ANOVA) [21] with gestational diet and measurement time-point as factors.

### Perfusions

Pups who were randomly selected for either P11.5 (N_*SD*_=14, N_*KD*_=18) or P21.5 (N_*SD*_=17, N_*KD*_=23) perfusion were initially sedated by an intraperitoneal injection (0.1 ml/10 gr of body weight) containing Ketamine and Xylazine, at concentrations of 150 mg/kg and 10 mg/kg, respectively. Mice were then trans-cardially perfused with 30mL of 0.1 M PBS containing 1 *μ*L/ml Heparin and 2 mM Gadolinium (“ProHance” gadoteridol by Bracco Diagnostics), followed by 30mL of 0.1 M PBS containing 4% Para-formaldehyde (PFA) and 2 mM Gd. Perfusion fluid flow rate was 100 mL/hr [22]. Following perfusion, the brain was extracted within the skull, but the lower jaw and skin were removed. The brain and remaining skull structure were incubated in 4% PFA containing 2 mM Gd overnight at 4 ^*o*^*C*, and then transferred to 0.1 M PBS containing 0.02% sodium azide with 2 mM Gd for at least 3 days prior to imaging.

### Brain imaging

Fixed brains were imaged by MRI with a 3D Diffusion Tensor Imaging (DTI) sequence consisting of a fast spin echo train length of 6 and a TR of 325 ms. TE for the first echo was 30ms, and then the echo spacing was set to 6 ms for the remaining 5 echoes. The diffusion parameters were as follows: *γ*=2.675∗10^8^*rad*/*s*▪*T*, *δ*=4 *ms*, and *Δ*=15 *ms*. The imaging field-of-view was 14 x 14 x 25 mm^3^, the matrix size was 108 x 108 x 192, and the image was acquired using 2 averages. This yielded a final image with 0.130 mm isotropic voxels. Five images with minimal diffusion weighting (b = 0 s/mm^2^) and 30 high b-value images (b = 1918 s/mm^2^, *G*=35 *G*/*cm*) were acquired according to the Jones30 scheme [23-25]. Total imaging time for this sequence was ∼14 hrs.

**Figure 1:**
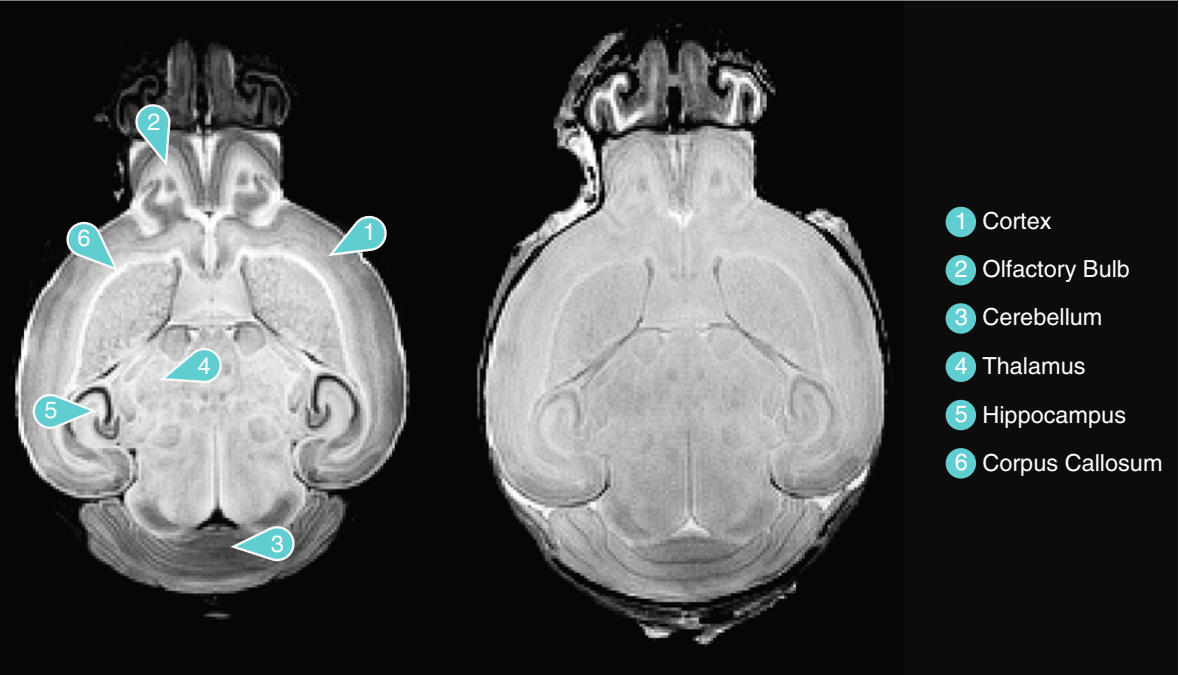
**An image of a neonatal mouse brain acquired with DTI, along with labelling of selected brain regions.** Left: the average image of the 30 high-b diffusion-weighted images of the same brain. Right: the b0 image.

An example of a single MR image of a mouse brain acquired with DTI is depicted in Figure 1, along with labelling of several regions. Figure 1 shows the b0 image (Right) along with the average image of the 30 high-b diffusion weighted images (Left), which was used for image analysis. As can be seen, the image obtained from averaging the high-b diffusion weighted images yields a significantly improved anatomical contrast.

### Image statistical analysis

**Figure 2:**
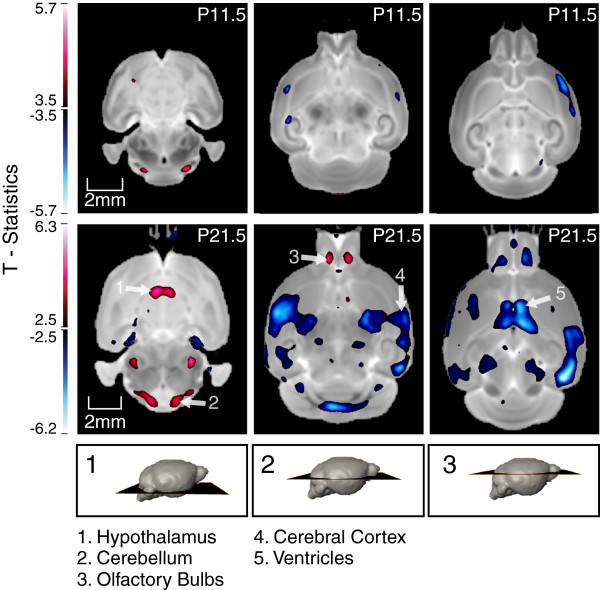
**T-statistics map overlaid on top of the final average registered P11.5 and P21.5 brain images, highlighting voxels with statistically different deformation (FDR ≤ 15%).** Blue regions are statistically smaller, whereas red regions are statistically larger in relative volume in the SD compared with the KD brain. Shown are three cross-sectional views of the average P11.5 brains, along with the corresponding cross-sections at P21.5, for comparison.

At each of the two time-points, an average of the 30 high b-value diffusion weighted images of the SD and KD pups were geometrically aligned with one another, and saved within a common coordinate system. The overlapping images were then used to construct a single consensus average image by first undergoing linear (6 parameter, followed by a 12 parameter) alignment through a series of rotations, translations, scales, and shears. Then, an iterative non-linear alignment procedure created local deformations in each image, thereby bringing all images into alignment. All registrations were performed using a combination of ANTS algorithm [26] and mni_autoreg tool [27]. The collection of all linear and non-linear deformations across each image formed a *deformation field*, which contained the transformation from each embryo image to the consensus average image. The Jacobian matrix was calculated for every point in the deformation field, and the determinant of the Jacobian was then calculated for each image voxel (i.e. 3D pixel). The log-transform of these determinants were computed, assigning positive values to the determinants that were >1 and negative values to those <1. Smoothing was applied to the resulting values using a 0.2mm FWHM Gaussian kernel, to improve normal distribution and facilitate the following t-statistics. Analysis of these results using t-statistics allowed to identify whether the KD group significantly differed from the SD group of brains in any particular 3D region. The results from this deformation-based analysis are reported as a structural brain image overlaid with the voxel’s t-statistics, as shown in Figure 2 by the red and blue scale-bars in regions that were significantly different. This analysis accounted for multiple comparisons through a False Discovery Rate (FDR) technique [28,29], as reported along with each image. To quantify whole-structure volume changes, brain regions were segmented from the final average image using a pre-existing atlas [30]. The inverse deformation field from the registration was then applied to back propagate the atlas onto the original unregistered images, allowing the regional volume differences to be calculated. Only regions that were significantly different at an FDR of <15% in this region-based analysis are reported in the results. FDR was calculated based on all 62 regions in the atlas.

## Results

### Lactation in KD Dams Leads to Fatal Ketoacidosis

In the initial trial phase, 4 KD dams remained with their biological litter post parturition, and during lactation. However, lactation appeared to cause severe physiological strain to all KD dams. Soon after parturition they started exhibiting signs of high stress, as was also apparent by them cannibalizing their litter. Measurements of their blood glucose and ketone revealed elevated values (Glucose: ∼20 mmol/L and Ketone: ∼6 mmol/L), indicating development of ketoacidosis. This ketoacidosis rapidly progressed to a fatal metabolic state, leading to the death of the KD dams within a few days post parturition. Investigation of the KD pups indicated that they were indeed consuming some milk, but that the maternal KD milk – either in terms of its quantity or nutritional value – was insufficient to support their growth, since they remained small for their age. To ensure viability of both the KD dams and pups post parturition, we altered the protocol by adding a fostering procedure at P2.5. This change also necessitated adding a group of SD-foster mice, who mated during the KD and SD mating days, and who adopted the pups from the study groups. The KD and SD dams remained on their respective diets following separation from their pups at P2.5, and their metabolic status was assessed every other day, as previously described, until P20.5.

### Effects on maternal fertility and metabolic status

Significant differences in fertility were noted between the SD and KD mice. At the end of the 2-week mating period, a larger percentage of the KD female mice remained non-pregnant compared with the SD female mice: 26% (N=31) versus 6% (N=17). This decrease in fertility was also accompanied by a smaller litter size, as noted at P2.5: 13±3 for the SD, versus 10±3 for the KD group (Mean ±Stdev ; N_*SD*_=46 ; N_*KD*_=25 ; p <0.01).

**Figure 3:**
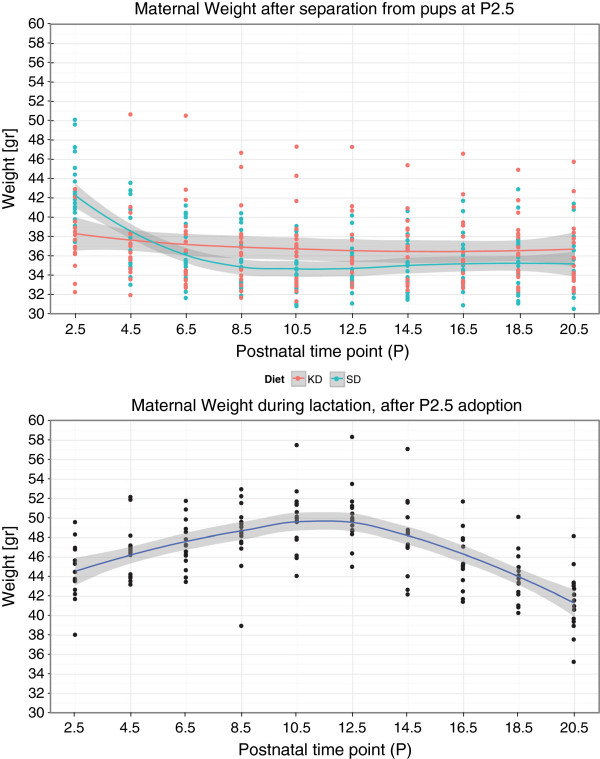
**Maternal weight between P2.5 and P20.5 in (Top) the KD and SD control dams who were separated from their pups at P2.5, and in (Bottom) the lactating SD foster dams.** Local regression is used to visualize the trend, where the shaded region indicates the standard error of the regression. (Top: N_*SD*_=19, N_*KD*_=17, Bottom: N_*Lactating*−*FosterDams*_=15 per time point).

Maternal KD body weight at P2.5 was also found to be statistically lower than the SD weight (p <0.0001) as illustrated in Figure 3 showing the change in maternal weight versus time between P2.5 and P20.5.

Figure 3 also reveals a notable difference in trends of body weight versus time between the dams that were separated from their litters at P2.5, and those who remained with their litters until P21.5. Separation from their litters soon after parturition resulted in a decrease in KD and SD-control body weight between P2.5 and P16.5, instead of a weight increase in mid-lactation, as observed in the lactating dams. At P2.5 the KD dams have a significantly lower body weight compared with SD control dams. Also, their decrease in weight between P2.5 and P20.5 is more gradual than the weight drop observed for the SD control dams. One speculation is that these two differences may be caused by under-developed mammary glands, and thus, reduced tissue volume that undergoes apoptosis during mammary involution.

**Figure 4:**
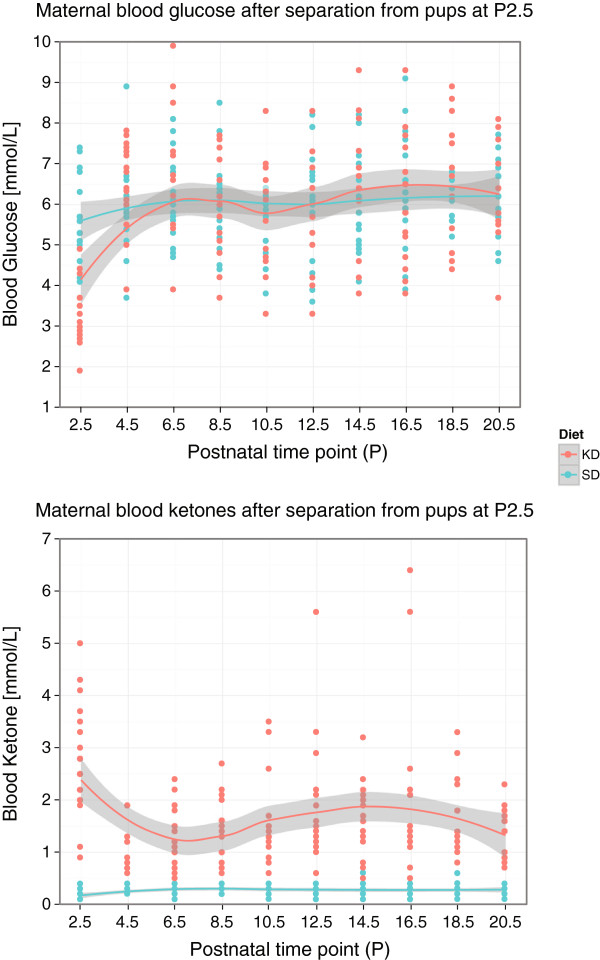
**Blood glucose and ketone concentration over time in KD and SD dams following separation from their litters at P2.5, and until P20.5.** Local regression is used to visualize trend, where the shaded region indicates the standard error. (N_*SD*_=19, N_*KD*_=17 per time point).

In addition, separation of the KD dams from their litters at P2.5 prevented ketoacidosis from developing. Their blood ketone concentration, which was elevated at P2.5, dropped to a concentration of 1-3 mmol/L by P4.5. These ketone concentration values correspond to average values during stable ketosis, as shown in our previous study [16]. Further, their blood glucose concentration stabilized by P6.5-8.5, and did not continue to increase past the normal range exhibited by the SD control dams. This data is shown in Figure 4.

In general, separation from the litters at P2.5 resulted in stabilization of ketosis and avoidance of ketoacidosis in the KD dams. At P20.5, all KD dams appeared normal.

### Physiological growth of adopted pups

**Figure 5:**
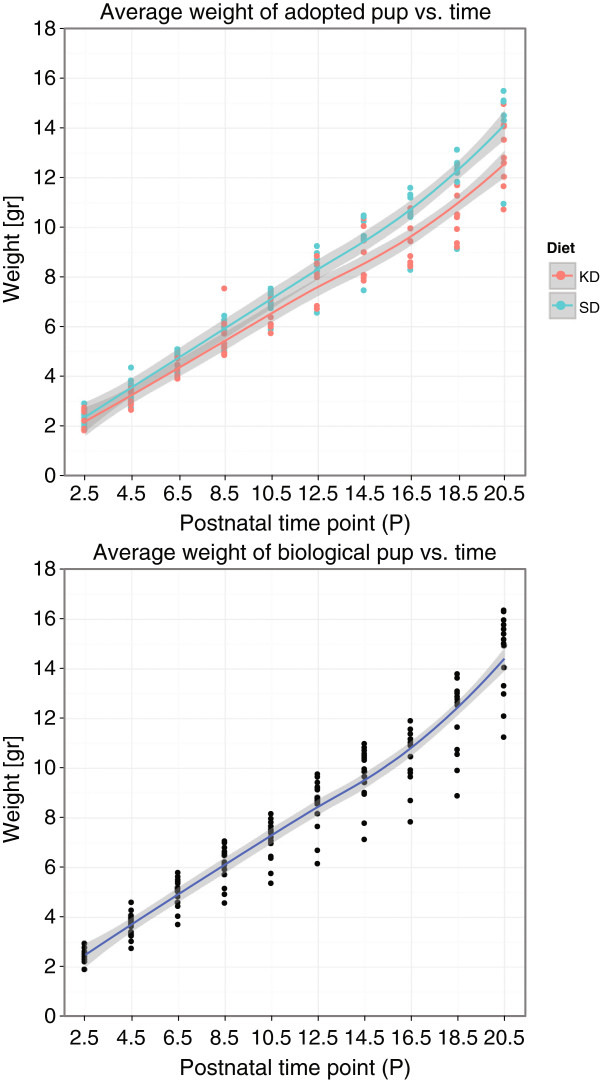
**Average pup weight per litter during lactation.** (Top) Adopted SD and KD and (Bottom) Biological SD pup weight during lactation, between P2.5 and P20.5 (Number of litters: N_*SD*−*Adopted*_ = 7, N_*KD*−*Adopted*_ = 8, N_*SD*−*Biological*_ = 15. Average number of pups per litter: N_*SD*−*Adopted*_ = 8, N_*KD*−*Adopted*_ = 8, N_*SD*−*Biological*_ = 5).

The body weight of adopted and biological pups throughout lactation is summarized in Figure 5. It indicates a slightly slower weight gain for the KD pups compared with the adopted SD pups, particularly after P12.5.

The final average KD pup weight at P20.5 was found to be significantly lower than that of the SD counterpart (p <0.0001), whereas the average adopted SD weight was similar in value to that of the average biological SD pup (p >0.05). This indicates that it was not the adoption process but the maternal diet during gestation that leads to the slower weight gain.

**Figure 6:**
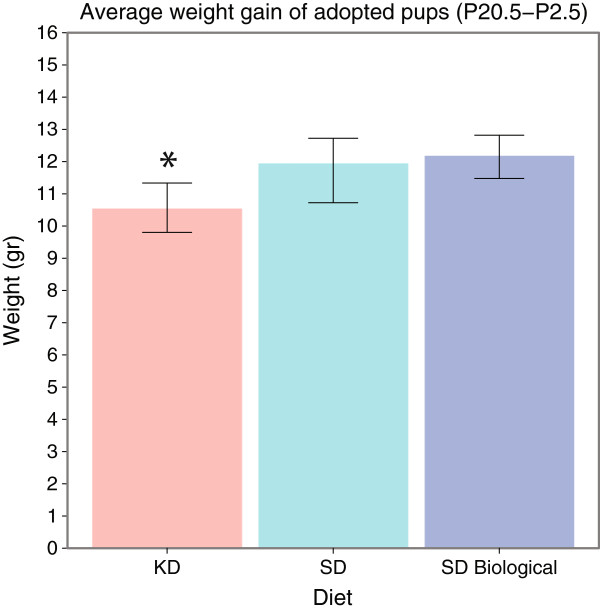
**Average weight-gain per litter of Adopted KD, Adopted SD, and Biological SD pups between P2.5 and P20.5.** (Number of litters: N_*SD*−*Adopted*_ = 7, N_*KD*−*Adopted*_ = 8, N_*SD*−*Biological*_ = 15).

The similarity between the weight gain of the adopted and biological SD pups is also evident in the average weight gain of the pups between P2.5 and P20.5, as show in Figure 6. Figure 6 further shows a smaller weight gain of the adopted KD pups compared with both the adopted and biological SD pups during the first 3 weeks of postnatal life (p <0.05).

### Brain structure at P11.5 and P21.5

A consensus average brain image was constructed at each of the two time-points. Analysis of overall brain volume indicated that the SD and KD brain did not statistically differ at either time point; however, at P21.5 there was a tendency for the KD brain to be smaller than the SD one [SD: 507±32 *mm*^3^, N_*SD*_=17 ; KD: 491±20 *mm*^3^, N_*KD*_=23 (Mean ±Stdev), p=0.053]. A similar statistical analysis but of voxel volume relative to the entire brain revealed several differences between the KD and SD brains. The voxel-wise analysis indicated that at P11.5 the KD brain had a slightly smaller cortex compared with the SD brain (FDR=15%). However, these differences are insignificant below an FDR=15%. At the later time-point, P21.5, even larger areas of the cortex showed relative reduction in volume at an FDR=15%, which also persisted at an FDR=5%. In addition, the organ-based analysis revealed that the corpus calosum, fimbria, lateral ventricles, and hippocampus were also relatively smaller in volume in the P21.5 KD compared with the SD brain. The hypothalamus as well as the medulla were found to be relatively enlarged in the KD compared with the SD. While some regions within the olfactory bulb were relatively enlarged in the KD brain compared with the SD counterpart, these did not result in a statistically significant difference in the relative volume of the entire structure following its segmentation. The morphological differences are shown in the three panels in Figure 2.

## Discussion

Our study first reveals that a gestational ketogenic diet decreases maternal fertility and litter size. This may be attributed to the lower dietary protein contents in the KD versus the SD. A study by Goettsch (1960) [31] indicated a direct relationship between protein consumption and maternal fertility, as measured by estrus cycle length. The same study also found that reduced dietary protein could lead to smaller litter size. A similar observation was made in a human study using a low carbohydrate, high protein diet, which revealed increased fertility with increasing protein intake [32]. However, other researchers have not observed a reduction in litter size as a result of a protein deficiency, per se [33]. These researchers speculated that during lactation the “females could compensate for a dietary protein deficit by reducing their reproductive effort to match the lower availability of protein” [32]. In our study, the smaller litter size was noted only at P2.5. Hence, it is possible that the litter size upon parturition was not statistically different between the study groups, and that the difference noted at P2.5 was due to maternal cannibalization of the KD pups. Such maternal behaviour could be caused by the high metabolic stress the KD dams were experiencing, and would indirectly reduce their reproductive efforts [34].

Our study also shows that KD dams are at a high risk for developing ketoacidosis during lactation. The elevated maternal serum ketone along with the statistically lower serum glucose concentration, as measured at P2.5, were the early signs of ketoacidosis and an indication of physiological strain due to lactation. Since lactation significantly raises the metabolic rate, to more than twice the normal level [35], this high metabolic demand may be the reason for the development of ketoacidosis in the KD dams. Cessation of milk production and mammary involution, as expected to be induced by separation from the litter at P2.5 [36,37], facilitated stabilization of the KD dams’ metabolism within 4 days. Their elevated ketone concentration at P2.5 dropped to below ∼2mmol/L, and their below-normal P2.5 glucose concentration increased to ∼6mmol/L, both of which were within the normal stable-ketosis values [38].

Change in maternal body weight over the first three weeks post parturition exhibited a different trend depending on whether the dams were breastfeeding. The weight of the breastfeeding foster SD dams increased gradually between P2.5 and P10.5, but then decreased until P20.5, when the offspring were weaned. On the other hand, the weight of the dams who were separated from their litters at P2.5 only decreased thereafter, and stabilized by around P14.5. In this case, there was no intermediate increase in body weight. In the case of both the SD-control and KD dams, their separation from the litter soon after parturition lead to milk stasis, cessation of milk production, along with mammary involution [36,39]. In fact, their pattern and timing of weight loss is consistent with those of mammary involution and mammary cell apoptosis, as reported by Richert et al. [40]. The difference in the rate of weight loss of the SD-control and KD dams may be attributed to differences in mammary tissue volume. The fact that the KD dams encountered metabolic difficulties lactating, also supports our speculation that their mammary glands were under-developed. Under-developed mammary glands have smaller milk storage capacity, leading to decreased production of milk [40,41]. The insufficient milk production is, in turn, supported by our observation of the retarded growth of the KD pups who originally remained with their biological KD mothers during lactation.

Offspring growth, as measured by the average weight gain of the pups, was also different in the adopted KD compared with the adopted SD groups. The KD pups exhibited a slower weight gain, which became particularly noticeable after P12.5, and resulted in a statistically significant difference in weight at P20.5 (p <0.05). This difference in weight gain could be attributed to the dietary mismatch the KD pups experienced between pre- and post-adoption periods. Prior to adoption, the KD pups received a high-fat diet through maternal KD milk; whereas post-adoption, they received a low-fat diet through SD milk. This change from abundance to sparsity in fat renders the postnatal, post-adoption phase a state of undernutrition. Since postnatal undernutrition has been shown to result in slower postnatal growth [42], this would explain the significantly lower body weight of the KD pups at P20.5. Another potential explanation is that the dietary mismatch rendered the KD pups weaker compared with the biological SD ones, limiting their access to maternal milk, and resulting in hindered growth. Overall, the early dietary mismatch is a confound that may play a role in the slowed growth of the KD pups betweem P12.5 and P21.5.

Offspring brain structure was also altered as a result of prenatal and early-postnatal exposure to a maternal KD. At P11.5, when compared with the SD brain, the KD brain had a slight bilateral enlargement of cortical volume, and unilateral reduction in mid-brain volume, when computed as a percentage of the total brain volume. At the later time-point, P21.5, the relative bilateral enlargement of the cortex became more prominent. In addition, the KD hypothalamus was also relatively enlarged; whereas the hippocampus, corpus callosum and olfactory bulb were smaller in relative volume. These changes could be due to the augmented prenatal nutritional environment of the KD mouse offspring. Since the mouse brain undergoes significant organizational processes in prenatal life, such as neurogenesis, neuronal migration, and differentiation, destabilization of this prenatal period is detrimental for proper brain development [43]. A metabolic insult that alters any of these processes can impact neuronal circuit formation and cerebral architectural organization, which would become apparent only during the brain’s postnatal growth spurt in the mouse [43]. We demonstrate that the prenatal exposure to a maternal KD is the cause of the observed structural alterations, confounded by the KD-to-SD switch at P2.5.

The exact effects of a maternal, gestational KD have not been thoroughly investigated. Thus far, it is known that ketones are natural by-products of fat metabolism [44], and can serve as metabolic fuels in lieu of glucose [12,45,46]. Yet, their yield during ATP hydrolysis is greater than that of glucose [4,5,47], suggesting ketosis provides a greater amount of energy compared with glycolysis. While the greater energy availability may promote cellular growth, it may be counteracted by ketones’ inhibition of pyrimidine synthesis [45], alteration of cerebral nucleotide bio-synthesis and of cerebral amino acid metabolism [13,48]. The overall effect of ketones on the developing brain, would then depend on the balance between these various factors. Specifically, a positive net effect could explain the increase in relative volume observed in the hypothalamus and medulla; whereas, a negative net effect could explain the relative decrease in volume observed in the hippocampus and corpus callosum.

Studies on maternal ketosis in the pig revealed an increase in weight, protein content and cell-size in the fetal pig brain [49]. Studies using the mouse showed that its brain preferentially utilizes ketones for energy, and as precursors of amino-acid and lipid synthesis [50,51]. Similar studies on the rat found elevated levels of the ketone *β*−hydroxybutyrate, particularly in the striatum and cortex, in late gestation and early postnatal period [52]. Together, these studies indicate that ketones do play a dominant role in prenatal brain growth. They also imply that an increase in ketone availability, as may occur during maternal KD could, in fact, alter the volume of various brain regions in the offspring, supporting our observations. The altered brain structure may be indicative of functional and behavioural changes in the KD offspring. The exact outcome remains to be elucidated by post-natal behavioural studies of the offspring. The genetic similarity between the mouse and the human suggests that our observations may resemble the potential impact on the human fetal brain, if exposed to a similar gestational ketogenic diet at the corresponding developmental time period.

## Conclusion

To reveal the potential impact of a gestational Ketogenic diet on brain structure in the offspring, we imaged neonatal brains from mice whose mothers were either fed a Standard Diet (SD) or a Ketogenic Diet (KD) during gestation. Our initial observations of the KD dams indicated reduction in maternal fertility and litter size, as well as a substantially increased risk of developing fatal ketoacidosis during lactation. To ensure viability of both the dams and their pups, we carried out fostering at P2.5. Following separation from their litters at P2.5, the KD dams exhibited a more gradual weight decrease, compared with the control SD dams. The average fostered KD pup exhibited retarded weight gain during the first 3 weeks postnatally, compared with the average fostered SD pup. Finally, assessment of brain structure revealed several major structural differences at both P11.5 and P21.5. The KD brain had a bilateral relative decrease in the cortex, hippocampus, corpus callosum, fimbria, and lateral ventricles, but a relative enlargement in volume in the hypothalamus and medulla. These anatomical differences may be the consequence of the altered nutritional environment during prenatal life, when significant organizational processes occur in the mouse brain. These changes could also be triggered by certain brain regions’ preference for ketones during development, as well as by the increased efficiency of energy production from ketones. In conclusion, these cerebral anatomical alterations could be indicative of underlying functional and behavioural changes which could become apparent in later postnatal life. Overall, our study points out the significance of prenatal nutrition, and indicates that it drives subsequent postnatal brain development, even when matching postnatal nutrition exists.

## Abbreviations

3D: Three Dimensional; DTI: Diffusion tensor imaging; FDR: False discovery rate; Gd: Gadolinium; KD: Ketogenic diet; MCTs: Monocarboxylate transporters; MRI: Magnetic resonance imaging; P: Postnatal day; PBS: Phosphate buffered saline; PFA: Para-formaldehyde.

## Competing interests

The authors declare that they have no competing interests.

## Authors’ contributions

DS and MH created the study protocol, supervised the study, and wrote the manuscript. DS conducted the study, collected the data, and analyzed it. DS and JE carried out the brain imaging. All authors read and approved the final manuscript.

## Pre-publication history

The pre-publication history for this paper can be accessed here:

http://www.biomedcentral.com/1471-2393/13/198/prepub
